# Deletion of LOX-1 Protects against Heart Failure Induced by Doxorubicin

**DOI:** 10.1371/journal.pone.0154994

**Published:** 2016-05-19

**Authors:** Chiharu Yokoyama, Takuma Aoyama, Takahiro Ido, Akemi Kakino, Takeru Shiraki, Toshiki Tanaka, Kazuhiko Nishigaki, Aiko Hasegawa, Yoshiko Fujita, Tatsuya Sawamura, Shinya Minatoguchi

**Affiliations:** 1 Department of Cardiology, Gifu University Graduate School of Medicine, Gifu, Japan; 2 Cardiovascular Center, Kizawa Memorial Hospital, Minokamo, Japan; 3 Department of Vascular Physiology, National Cerebral and Cardiovascular Center Research Institute, Suita, Osaka, Japan; 4 Department of Physiology, Shinshu University School of Medicine, Matsumoto, Japan; Virginia Commonwealth University, UNITED STATES

## Abstract

Oxidative stress is one of the major factors in doxorubicin (DOX)-induced cardiomyopathy. Lectin-like oxidized low-density lipoprotein (oxLDL) receptor-1 (LOX-1) plays an important role to regulate cardiac remodeling and oxidative stress after ischemia-reperfusion. Therefore, we examined whether or not LOX-1 contributes to the pathogenesis of DOX-induced cardiomyopathy. Cardiomyopathy was induced by a single intraperitoneal injection of DOX into wild-type (WT) mice and LOX-1 knockout (KO) mice. Echocardiography and catheter-based hemodynamic assessment apparently revealed preserved left ventricular (LV) fractional shortening (FS) and cavity size of LOX-1 KO mice compared with those of WT mice after DOX administration. Less production of tumor necrosis factor alpha (TNF-α) and interleukin-1 beta (IL-1ß) was observed in LOX-1 KO mice than WT mice after DOX administration. Western blotting analysis also showed lower activation of nuclear factor κB (NF-κB) and p38 mitogen-activated protein kinase (MAPK) in LOX-1 KO mice treated with DOX than WT mice treated with DOX. In fact, NF-κB-dependent gene expressions of LOX-1 and vascular cell adhesion molecule-1 (VCAM-1) were suppressed in LOX-1 KO mice treated with DOX compared with WT mice treated with DOX. Therefore, histological analyses showed attenuation of leukocyte infiltration and cardiac fibrosis in LOX-1 KO mice compared with WT mice. Meanwhile, extracellular signal-regulated kinase MAPK (ERK) inactivation and decreased expression of sarcomeric proteins and related transcription factor GATA-4 in WT mice treated with DOX administration were not seen in LOX-1 KO mice treated with DOX administration and WT and LOX-1 KO mice treated with vehicle. Decreased expression of sarcometric proteins resulted in smaller diameters of cardiomyocytes in WT mice than in LOX-1 KO mice after DOX treatment. The expression of LOX-1 in cardiomyocytes was much more abundant than that in endothelial cells, fibroblasts and inflammatory cells. Endothelial cells, fibroblasts and inflammatory cells treated with DOX showed no elevated LOX-1 expression compared with those treated with vehicle. However, cardiomyocytes treated with DOX showed much more expression of LOX-1 than those treated with vehicle. Immunohistochemistry study also showed that LOX-1 expression was strongly elevated in cardiomyocytes in the heart tissue of mice treated with DOX *in vivo*. We conclude that LOX-1 in cardiomyocytes plays the most important roles in the pathology of DOX-induced cardiomyopathy. LOX-1 deletion altered the LOX-1-related signaling pathway, which led to improvements in cardiac function, myocardial inflammation, fibrosis and degenerative changes after DOX treatment.

## Introduction

The lectin-like oxidized low-density-lipoprotein receptor (LOX-1), which belongs to the type D scavenger receptors, was initially cloned from aortic endothelial cells in 1997 [1–3]. LOX-1 is considered to be the major receptor for oxLDL in human vascular endothelial cells and is also expressed in intimal smooth muscle cells and lipid-laden macrophages in human atherosclerotic lesions, as well as in plaque neovasculature [4–10]. LOX-1 plays a role in oxLDL-induced apoptosis of vascular smooth muscle cells and in the production of matrix metalloproteinases, which can cause plaque rupture and lead to acute coronary syndrome [11–16]. Soluble LOX-1 cleaved within the cytoplasmic domain of the cell membrane was found to be significantly elevated in patients with acute coronary syndrome and stable coronary artery disease [17–19]. Moreover, we have recently found LOX-1 plays an important role in ischemia-induced angiogenesis and reported this novel physiological function of LOX-1 [20].

Although the role of LOX-1 as an atherosclerosis or a neovasculature-related molecule has gradually become clearer, its pathophysiological function in the hearts has not been fully described. The role of LOX-1 after remodeling following ischemia–reperfusion (I–R) was been investigated by other groups [12, 21]. They reported that significantly less necrosis in LOX-1 KO mice was found in WT mice after I-R in the hearts.

This study provides evidence for a critical role of LOX-1 in myocardial I–R injury. Reperfusion injury is associated with the production of large amounts of reactive oxygen species (ROS), which can modify LDL to an oxidized form, oxLDL, suggesting that LOX-1 is activated by oxLDL and promotes the infiltration of inflammatory cells into the hearts. Therefore, LOX-1 exacerbates I-R injury via a redox-sensitive reaction.

LOX-1 has been reported to play an important role in congestive heart failure as well [22]. LOX-1 expression was markedly upregulated in the LV of salt-sensitive Dahl rats with heart failure and correlated with decreased ejection fraction (EF) and increased brain natriuretic peptide (BNP) [23]. These findings suggest that the LV expression of LOX-1 can serve as a novel biomarker of heart failure in hypertensive heart disease. Thus, a marked increase in the LV expression of LOX-1 in failing heart may significantly contribute to increased serum levels of soluble LOX-1 (sLOX-1). However, it has remained unknown whether or not activation of LOX-1 in the hearts is a cause or result of heart failure.

DOX is well known as a highly effective anticancer agent but its potent cardiotoxicity leading to DOX-induced cardiomyopathy causes the limitation of the clinical use of DOX [24–27]. Conventional heart failure therapy including diuretics, renin–angiotensin–aldosterone system inhibitors, ß-blockers and so on has been shown to attenuate DOX-induced cardiomyopathy and heart failure but the only viable choice for patients with serious DOX-induced cardiomyopathy and heart failure is heart transplantation [28]. It is notable that cytokine release mediated by activation of the innate immune system is believed to be involved in the pathogenesis of DOX-induced cardiotoxicity [29, 30]. As LOX-1 is closely related to inflammation, we assumed that LOX-1 is also involved in the development of DOX-induced cardiomyopathy, as has been shown in TLR-2 and TLR-4 deficient mice [27, 31]. The pathophysiological role of LOX-1 in DOX-induced cardiomyopathy has not yet been investigated. Therefore, we studied whether or not LOX-1 deletion has an impact on the development of DOX-induced cardiomyopathy, including its functional consequences and mechanisms.

## Materials and Methods

### Cell culture

Neonatal cardiomyocytes and cardiac fibroblasts were prepared from 1- to 2-day-old mice as described previously [32, 33]. Isolated cardiomyocytes and passaged fibroblasts were cultured in DMEM containing 10% fetal calf serum for 2 days. Before each experiment, all cells were placed in serum-free DMEM for 24 hours and then were treated with vehicle or DOX (1 μM) for 9 hours. All cells were subjected to Western blotting or microscopic morphological examination. Murine coronary endothelial cells were obtained from Cell Biologics Inc. and passaged and cultured onto 96-well plates and 60-mm dishes in Basal Medium (Cell Biologics) containing 10% fetal calf serum and growth factor (VEGF, ECGS, HGF and heparin) for 2 days. Before treatment, endothelial cells were starved in serum-free medium and then treated along with the cardiomyocytes.

### Animals and treatments

LOX-1 KO mice with a C57/BL6J background were produced as described previously [20] and LOX-1 KO mice and control wild-type (WT) C57/BL6J strain mice were a kind gift from Dr. Tatsuya Sawamura (National Cerebral and Cardiovascular Center, Suita, Japan/ Department of Physiology, Shinshu University School of Medicine, Matsumoto, Japan). Male LOX-1 KO mice and WT mice were used at the age of 12 weeks. Mice were housed in a facility with a 12-hour/12-hour light/dark cycle and were given free access to water and standard rodent chow. The room was kept specific-pathogen-free. DOX HCl (Sigma Chemical Co.) was dissolved in saline and administered by intraperitoneal injection at a dose of 20 mg/kg. This study was carried out in strict accordance with the recommendations in the Guide for the Care and Use of Laboratory Animals of the National Institutes of Health (NIH Publication, 8th Edition, 2011). The protocol was approved by the Institutional Animal Research Committee of Gifu University (Permit Number: 17−68).

### Physiological studies

Mice were anesthetized via nasal mask with halothane (induction, 2%; maintenance, 0.5%) in a mixture of N_2_O and O_2_ (0.5L/min each). Then we performed transthoracic echocardiography on the 0 day prior to days 7, 14, 21 and 28 after DOX injection using a VISUAL SONICS Vevo 770 Imaging System. Left ventricular (LV) internal dimensions at end-diastole (LVDd) and at end-systole (LVDs) were measured digitally using the M-mode tracings and were averaged from 3 cardiac cycles. LV fractional shortening (FS) was calculated as [(LVDd-LVDs)/LVDd] x100. At day 14 after DOX administration, mice were anaesthetized as described above and their right carotid arteries were subsequently cannulated with microtip catheters (1.2Fr, Science Inc.) and advanced into the aorta and then into the left ventricle for recording LV systolic pressure (LVSP), LV end-diastolic pressure (LVEDP), maximal rate of pressure development (+dp/dt) and maximal rate of pressure relaxation (-dp/dt) of LV pressure.

### Morphological examination

At 2 weeks after DOX injection (the age of 14 weeks), mice were sacrificed by cervical dislocation following exposure to 2% halothane in a mixture of N_2_O and O_2_ (0.5 L/min each) until righting reflex was lost. Then their hearts were excised and subjected to further analyses such as morphological examination and Western blotting. For morphological examination, hearts were fixed with a 4% solution of paraformaldehyde in PBS and then embedded in paraffin and serially cut from the apex to the base. Sections were stained with hematoxylin and eosin (HE) and Sirius Red. For quantification of % fibrosis identified by Sirius Red staining, we analyzed the stained sections using a video system equipped with the image analysis system Luzek AP (NIRECO). The Sirius-Red-stained area could thereby be quantified relative to the total tissue area.

For immunostaining analysis, LOX-1 in the heart tissue of WT mice treated with vehicle or DOX was stained with anti-LOX-1 antibody (abcam) using immunoperoxidase staining.

Leukocytes were stained with CD45 antibody (BD Biosciences) using immunoperoxidase staining to evaluate leukocyte infiltration and its quantification was performed as described below. Quantitative assessments, including of fibrotic area and the number of CD45-positive infiltrating leukocytes, were carried out using this video system with 20 randomly chosen high-power fields (HPFs) in each heart (four groups of eight hearts each). Twenty cardiomyocytes/heart (four groups of eight hearts each) were randomly selected and the average transverse diameter of cardiomyocyte was calculated for quantitative analysis of cardiomyocyte size.

For immunofluorescence study of LOX-1, the cells (cardiomyocytes, endothelial cells and fibroblasts) were fixed using 4% paraformaldehyde in PBS pH 7.4 for 10 min at room temperature and treated for 10 min with PBS containing either 0.1% Triton X-100 and incubated with anti-LOX-1 antibody (abcam) for 2 hours at RT and subsequently stained with Alexa Fluor 488-conjugated anti-rabbit IgG (Cell Signaling) for 1 hour at room temperature. Cardiomyocytes were identified with acti-stain 555 phalloidin staining (Cytoskeleton). Fibroblasts were identified with anti-vimentin antibody labeled with Alexa Fluor 555 (Cell Signaling). Endothelial cells were identified with anti-CD31 and relevant secondary antibodies were labeled with Alexa Fluor 555.

### Flow Cytometry analysis

The peripheral blood samples were collected into heparinized tubes from the mice 6 hours after DOX injection. To remove erythrocytes, the blood samples were incubated in 0.165M NH_4_Cl for 15 min for hemolysis. Thereafter, the hemolyzed samples were washed in FACS-buffer (1 mg/mL BSA, 0.05% NaN_3_ in PBS), then incubated with anti-CD16/32 (2.4G2) (Fc-block, TONBO biosciences) and stained with fluorescence-labeled antibodies. Stained cells were analyzed with FACSCanto, and the resulting data was processed by using FlowJo software (Tree Star, Inc.). Leukocytes were identified with APC-conjugated anti-mouse CD45 antibody (BD Biosciences). Leucocyte subclasses, lymphocytes, monocytes, and polymorphonuclear cells (neutrophils as the dominant population), were selected by usual FSC and SSC gating. Detection of LOX-1 was performed by staining with PE-conjugated anti-mouse LOX-1 (R&D Systems).

### Proinflammatory cytokine production

The heart tissues from WT and LOX-1 KO mice were excised and rinsed in PBS. Tissues were homogenized in buffer containing 0.1% Igepal, centrifuged and supernatants were collected [34] and assayed by a commercially available ELISA (R&D Systems) for murine TNF-α and IL-1ß according to the manufacturer’s instructions.

### Measurement of ROS generation

Tissue (100 mg) was washed with saline to remove as much blood as possible. We blotted the tissue with paper towels and then measured its weight. 500 μl sucrose buffer (0.25 M sucrose, 10 mM Tris, 1 mM EDTA, pH 7.4) was added and the sample was homogenized by using Teflon homogenizer and then centrifuged at 10,000 g for 60 min at 4°C, and the supernatant was transferred to a new tube. ROS generation was detected using the SOD Assay Kit-WST (Dojindo Molecular Technology, Japan) in a microplate reader according to manufacturer's protocol.

### Western blotting

Expressions of LOX-1, VCAM-1 (abcam), GATA-4, MHC and troponin-I (Santa Cruz Biotechnology), total or phosphorylated p65 subunit of NF-κB, MAPKs, Akt (Cell Signaling), were assessed by Western blotting [20]. The blots were visualized using chemiluminescence (LumiGLO, Cell Signaling), and the signals of the bands were quantified with a Calibrated Imaging Densitometer (LAS-3000IR; FUJIFILM).

### Statistical analysis

All data are expressed as mean±SEM.

To test the significance of the differences in variance, the F-test or the Bartlett’s test were used. For analysis of nonparametric histological data, the Mann-Whitney test or Kruskal-Wallis test with Dunn’s post-test analysis were used. For other parametric analyses such as echocardiographic data, Student’s *t*-test, 1-way and 2-way ANOVA test with Bonferroni’s post-test analysis were used for statistical significance as appropriate.

Statistical significance was accepted at a value of P<0.05.

## Results

**Fig 1:**
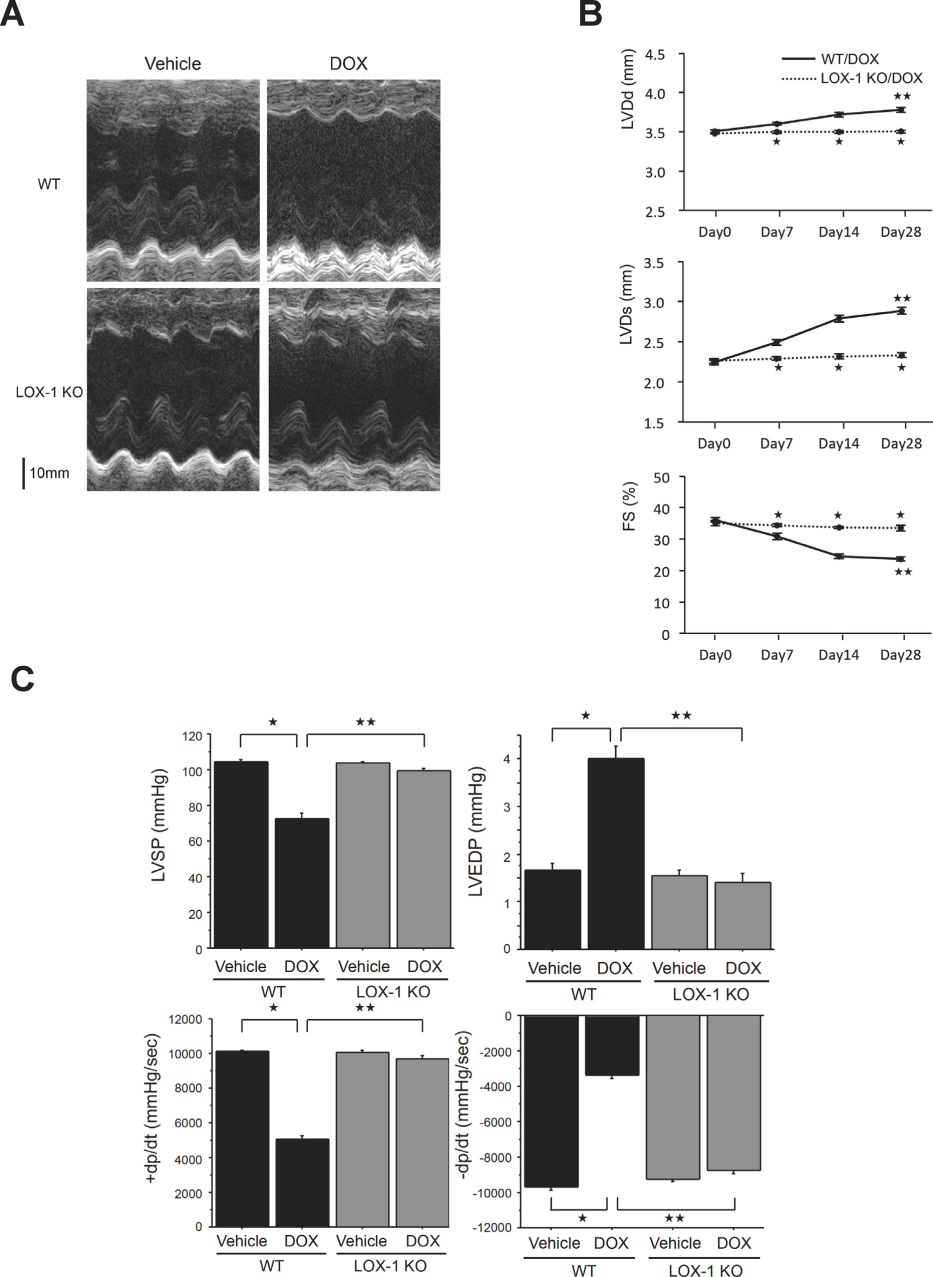
Assessment by transthoracic echocardiography and Millar micro-tip catheter transducer of the effect of LOX-1 deletion on LV dysfunction caused by DOX. A: Representative echocardiographic images for each of the four experimental groups. B: Time-dependent change of LV end-diastolic diameter (LVDd), LV end-systolic diameter (LVDs) and LV fractional shortening (FS) assessed by echocardiography in WT/DOX and LOX-1/DOX. Presented values are mean±SEM. ^★^ P<0.01, WT/DOX vs. LOX-1 KO/DOX, ^★★^ P<0.01, Day 0 vs. Day 28, n = 8. C: Effect of LOX-1 deletion on LV systolic pressure (LVSP), LV end-diastolic pressure (LVEDP), maximal rate of pressure development (+dp/dt) and maximal rate of pressure relaxation (-dp/dt) in the four experimental groups. Presented values are mean±SEM. ^★^P<0.01, WT/Vehicle vs. WT/DOX, ^★★^P<0.01, WT/DOX vs. LOX-1 KO/DOX, n = 8; day14

Both WT and LOX-1 KO mice were administered 20 mg/kg DOX or vehicle by intraperitoneal injection to investigate the effect of LOX-1 deletion on LV function after DOX treatment. LV function of WT mice was significantly impaired after DOX treatment. M-mode echocardiography demonstrated that LV chamber diameters of WT mice were significantly dilated compared with those of LOX-1 KO mice at 28 days after DOX administration, but there were no differences in LV chamber diameters between WT mice and LOX-1 KO mice treated with vehicle (Fig 1A). Echocardiography showed that all of LVDd, LVDs and FS were similar between WT mice and LOX-1 KO mice before DOX administration on Day 0 (Fig 1B). Administration of DOX progressively caused LV dilatation and worsening of LV systolic function of WT mice until Day 28 after DOX administration, but LOX-1 KO mice preserved LVDd, LVDs and FS compared with WT mice after DOX treatment. LV dilatation and systolic dysfunction were significantly suppressed in LOX-1 KO mice compared with WT mice (Fig 1B). Furthermore, DOX treatment did not alter LV dimension and systolic function of LOX-1 KO mice between Day 0 and Day 28 (Fig 1B). Table 1 and Fig 1B showed that parameters of LV dimension and function (LVDd, LVDs, IVSd, PWd, FS, SV, CO and LV mass) of WT and LOX-1 KO mice at Day 28 after DOX or vehicle administration. Only WT mice treated with DOX presented larger LVDd and Ds, smaller FS, SV and CO, and thinner IVSd and PWd relative to the other three groups of mice, but HR and LV mass of WT mice treated with DOX were not different from those of the other three groups of mice (Table 1).

**Table 1:** LV functional parameters (Led, LVDs, IVSd, PWd, FS, SV, CO and LV mass) in WT mice and LOX-1 KO mice at Day 28 after DOX administration.

		WT Vehicle	WT DOX	LOX-1KO Vehicle	LOX-1 KO DOX
HR	(/bpm)	417±8.61	396±5.63	409±11.95	395±8.40
LVDd	(mm)	3.51±0.016	3.78±0.032^a^	3.48±0.010	3.51±0.018
LVDs	(mm)	2.24±0.032	2.89±0.041^a^	2.26±0.028	2.33±0.032
IVSd	(mm)	0.66±0.013	0.54±0.017^a^	0.65±0.020	0.63±0.019
PWd	(mm)	0.69±0.010	0.59±0.015^a^	0.73±0.021	0.70±0.028
FS	(%)	36.14±0.721	23.70±0.628^a^	35.07±0.785	33.53±0.903
SV	(μl)	34.20±0.345	29.36±0.640^a^	32.80±0.553	32.33±0.718
CO	(ml)	14.28±0.402	11.63±0.245^a^	13.40±0.430	12.77±0.401
LVmass	(mg)	60.06±1.175	56.69±1.543	60.72±2.199	58.56±2.459

^a^P<0.01, WT/Vehicle vs. WT/DOX, n = 8

LV, Left ventricular; HR; heart rate; LVDd, LV internal dimensions at end-diastole; LVDs, LV internal dimensions at end-systole; IVSd, interventricular septal thickness at end-diastole; PWd, posterior wall thickness at end-diastole; FS, fractional shortening; SV, stroke volume; CO, cardiac output

Next, we confirmed the results of echocardiographic analysis by catheter-based hemodynamic measurements in the four groups of mice at 14 days after the administration of DOX (Fig 1C). Similar results with echocardiography were obtained by the invasive measurements with Millar catheter. LV hemodynamic parameters were assessed by pressure measurements in vivo to obtain LVSP, LVEDP, LV +dp/dt and LV -dp/dt. Fig 1C presents the effects of LOX-1 deletion on LVSP, LVEDP, LV +dp/dt and LV -dp/dt in WT and LOX-1 KO mice treated with vehicle or DOX. LVSP and +dp/dt in WT mice treated with DOX were significantly reduced compared with those in WT mice treated with vehicle, which is the baseline. LVEDP and–dp/dt in WT mice treated with DOX were significantly increased compared with those in WT mice treated with vehicle. On the other hand, all four parameters of LV cardiac function in LOX-1 KO mice treated with DOX were preserved at levels similar to the baseline, namely, WT mice treated with vehicle. LOX-1 KO mice treated with DOX had a significantly greater increase in LVSP and +dp/dt, less decline in −dp/dt and much less increase in LVEDP compared with WT mice treated with DOX (Fig 1C). Therefore, systolic and diastolic LV dysfunction induced by DOX was significantly improved by LOX-1 deletion.

**Fig 2:**
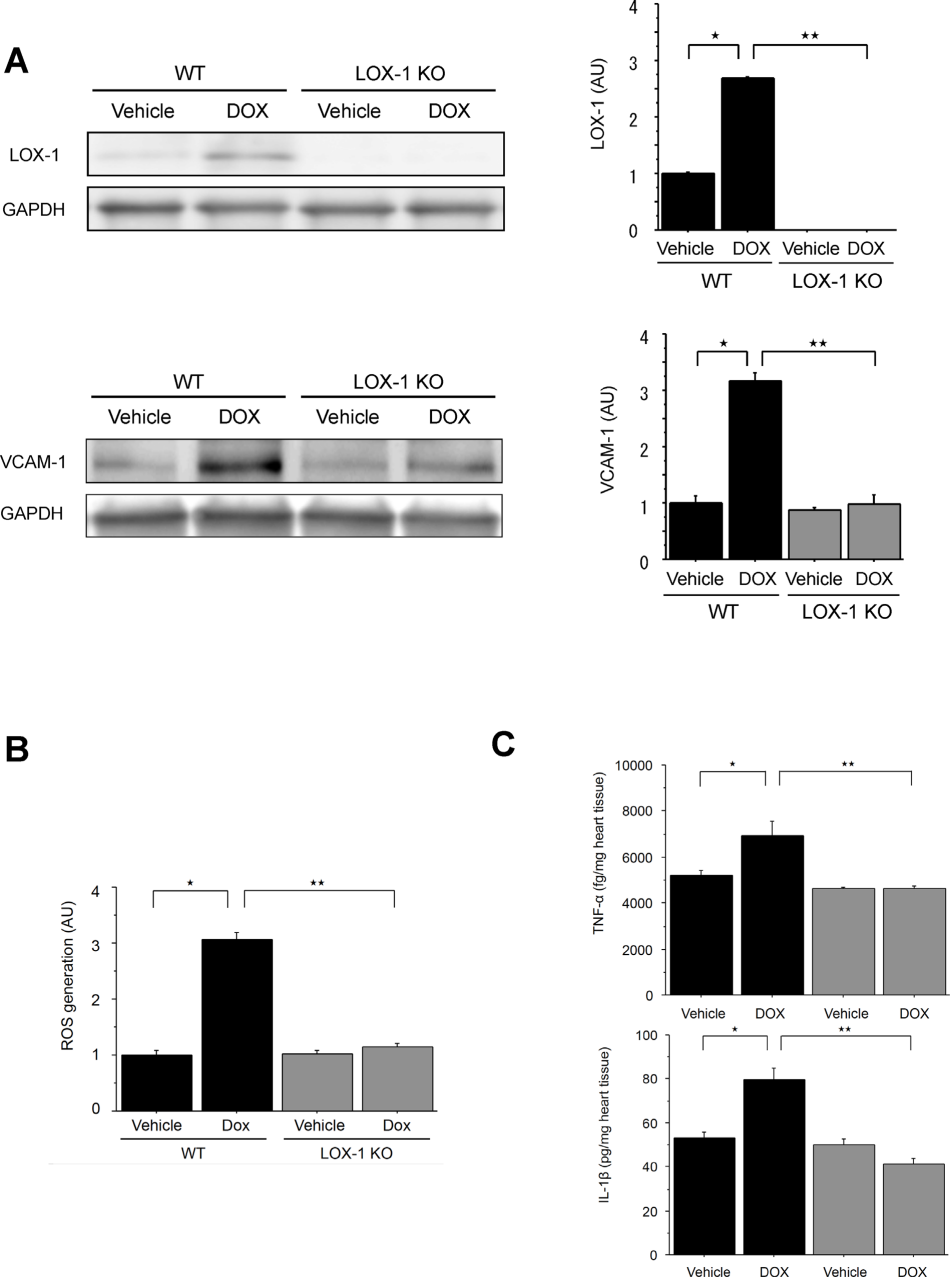
Effect of LOX-1 deletion on expressions of LOX-1 and VCAM-1 and productions of ROS, TNF-α and IL-1ß in the hearts after DOX treatment. A: Representative Western blot and quantification of LOX-1 and VCAM-1 in the four experimental groups. GAPDH is the internal control. Presented values (AU: arbitrary units) are mean±SEM. ^★^P<0.01, WT/Vehicle vs. WT/DOX, ^★★^P<0.01, WT/DOX vs. LOX-1 KO/DOX, n = 4. B: Measurement of ROS generation in the four experimental groups. Presented values (AU: arbitrary unit) are mean±SEM. ^★^P<0.01, WT/Vehicle vs. WT/DOX, ^★★^P<0.01, WT/DOX vs. LOX-1 KO/DOX, n = 8; day14. C: Quantification of cytokine production: TNF-α and IL-1ß in the four experimental groups. Presented values (fg/mg or pg/mg in the heart tissue) are mean±SEM. ^★^P<0.01, WT/Vehicle vs. WT/DOX, ^★★^P<0.01, WT/DOX vs. LOX-1 KO/DOX, n = 4; day14.

In order to examine whether or not LOX-1 and VCAM-1, both of which could be adhesion molecules for leukocytes and enhance inflammation, are upregulated in the hearts treated with DOX, we performed Western blot analysis. Western blot analysis showed that protein expressions of LOX-1 and VCAM-1, which are the NF-κB dependent inducible genes, in DOX-treated hearts of WT mice were significantly elevated compared with those in vehicle-treated hearts of WT mice (Fig 2A).

We simultaneously examined the expression levels of LOX-1 and VCAM-1 in vehicle- or DOX-treated hearts of two kinds of mice. As shown in Fig 2A, LOX-1 protein in LOX-1 KO mice was not detected in Western blotting at all and LOX-1 and VCAM-1 expression levels in DOX-treated hearts of LOX-1 KO mice were significantly less compared with those of WT mice.

DOX increases ROS production which has been implicated in the associated cardiotoxicity and ROS sources include the mitochondrial electron transport chain, xanthine oxidase, dysfunctional NOS and NADPH oxidase [35]. Activated LOX-1 enhances ROS production via Nox2 NADPH oxidase [20, 36]. Therefore, we measured ROS generation in the hearts after DOX or vehicle treatment by using the SOD Assay Kit.

ROS generation was significantly enhanced in the DOX-treated hearts of WT mice compared with the vehicle-treated hearts of WT mice (Fig 2B). On the other hand, ROS generation in the DOX-treated hearts of LOX-1 KO mice was significantly lower than that of WT mice (Fig 2B).

Proinflammatory mediators such as TNF-α and IL-1ß are important in the pathogenesis of chronic heart failure [37–39]. We performed ELISA assay to evaluate the productions of TNF-α and IL-1ß in the hearts of WT and LOX-1 KO mice treated with vehicle or DOX. The ELISA assay showed significant increases of TNF-α and IL-1ß in the hearts of WT mice treated with DOX compared with those of the hearts of WT mice treated with vehicle (Fig 2C). On the other hand, the increases of TNF-α and IL-1ß in the hearts of WT mice treated with DOX were suppressed in the hearts of LOX-1 KO mice treated with DOX (Fig 2C).

**Fig 3:**
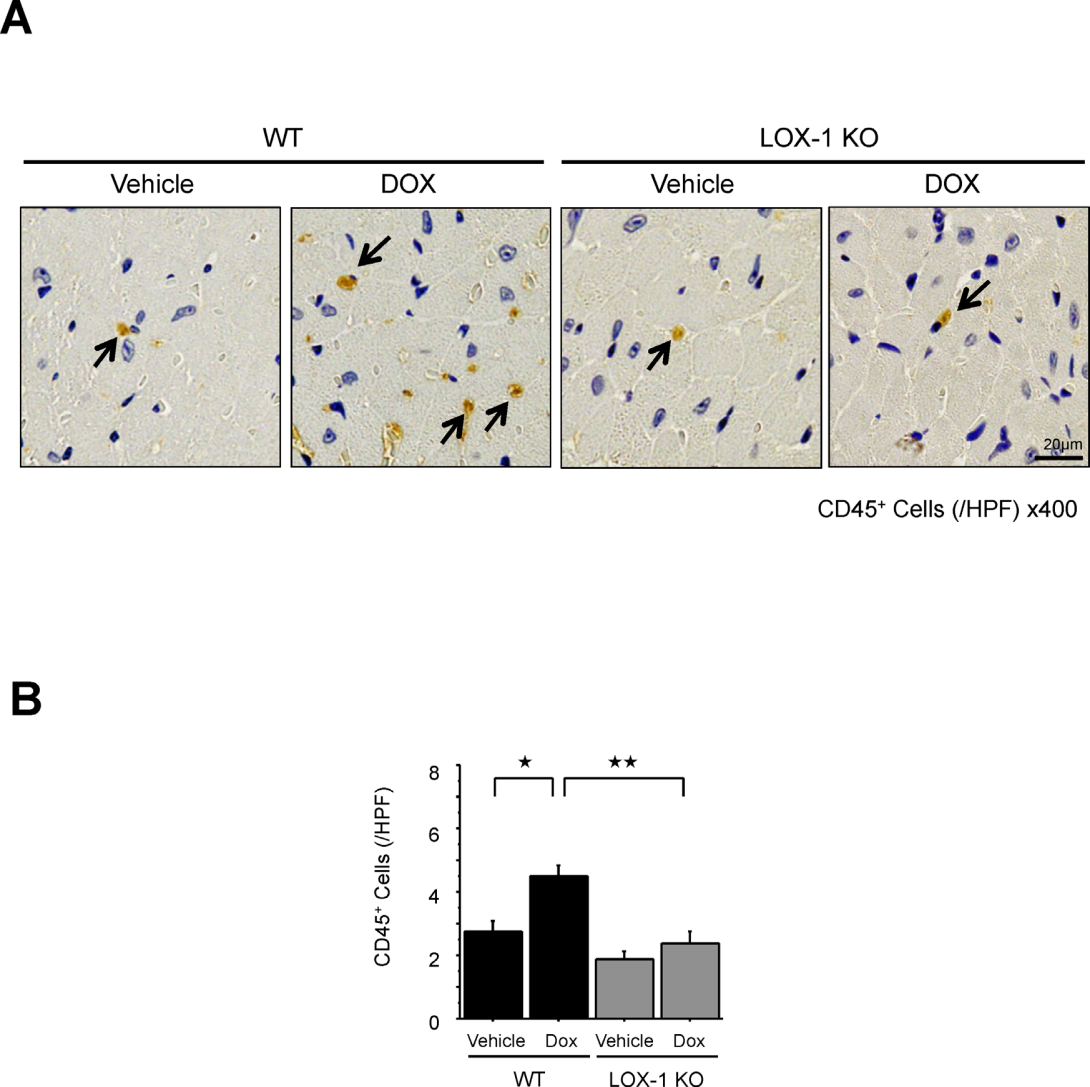
CD45-positive leukocyte infiltration in the four experimental groups. A: Representative images of infiltration of CD45-positive leukocytes (arrow: brown) in myocardium. Arrows indicate the brown-coloured strains of CD45-positive leukocytes. Representative scaled photographic images are shown (scale bars: 20 μm). B: Quantification of CD45-positive leukocytes in the four experimental groups (numbers of CD45-positive cells per high-power field; HPF, x400, scale bars: 20 μm) Presented values (/HPF) are mean±SEM. ^★^P<0.05 WT/Vehicle vs. WT/DOX, ^★★^P<0.05, WT/DOX vs. LOX-1 KO/DOX, n = 160; day14.

To evaluate how inflammatory cells are associated with DOX-induced cardiomyopathy, we performed immunohistochemistry to detect inflammatory leukocytes, which are CD45-positive cells, in the heart tissues, as shown in Fig 3A. Interestingly, WT mice treated with DOX showed more leukocyte infiltration in the heart tissues than WT mice treated with vehicle and there was no difference in leukocyte recruitment between WT mice and LOX-1 KO mice treated with vehicle (Fig 3B).

On the other hand, inflammatory leukocytes in the hearts of LOX-1 KO mice were significantly suppressed compared with those of WT mice after the administration of DOX (Fig 3B).

We performed histological analyses, namely, hematoxylin and eosin (HE) staining and Sirius Red, to evaluate cardiomyocyte cell size and cardiac fibrosis.

**Fig 4:**
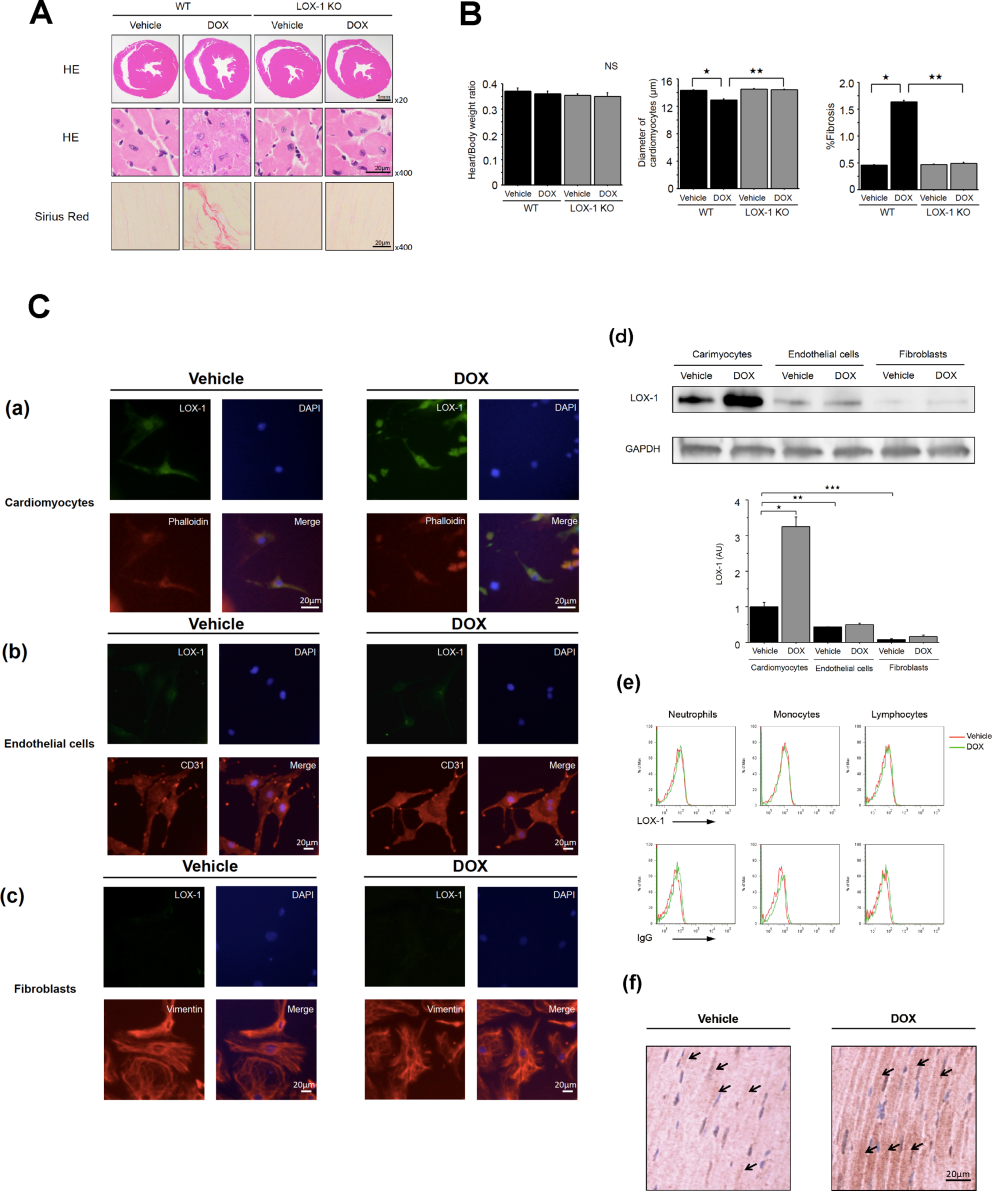
Histopathologic findings in the four experimental groups and evaluation of LOX-1 expression in cardiomyocytes, endothelial cells, fibroblasts and inflammatory cells. A: Photographs of the histological and immunohistochemical preparations. Top: Horizontal slices of whole ventricles stained with HE (x 20), Middle: HE (x 400), Bottom: Sirius Red (x 400). Representative scaled photographic images are shown. B: Quantitative analysis of heart/body weight ratio, diameter of cardiomyocytes and % fibrosis in the four experimental groups. Presented values are mean±SEM. ^★^P<0.01, WT/Vehicle vs. WT/DOX, ^★★^P<0.01, WT/DOX vs. LOX-1 KO/DOX, n = 160; day14, Male LOX-1 KO mice and WT mice were used at the age of 14 weeks. C: Histological analysis and Western blotting to evaluate LOX-1 expression. Immunofluorescence images of LOX-1 expression in (a) cardiomyocytes, (b) endothelial cells and (c) fibroblasts, which were treated with vehicle or DOX. LOX-1 (green), cardiomyocyte marker: phalloidin (red), endothelial cell marker: CD31 (red), fibroblast marker: vimentin (red), nuclei: DAPI (blue). Representative scaled photographic images are shown (scale bars: 20 μm). (d) Representative Western blot and quantification of LOX-1 in the above cells in the four experimental groups. GAPDH is the internal control. Presented values (AU: arbitrary units) are mean±SEM. ^★^P<0.01, Cardiomyocytes/Vehicle vs. Cardiomyocytes/DOX, ^★★^P<0.01, Cardiomyocytes/Vehicle vs. Endothelial cells/Vehicle, ^★★★^P<0.01, Cardiomyocytes/Vehicle vs. Fibroblasts/Vehicle, n = 4. (e) Flow cytometry analysis of the surface expression of LOX-1 on CD45-positive cells (neutrophil, monocyte and lymphocyte) in the peripheral blood of mice treated with vehicle or DOX. Plots show anti-LOX-1 Ab staining profile in the top panel and isotype control IgG staining profile in the bottom panel. Data are representative of five independent experiments (LOX-1 expression: ns, Vehicle vs. DOX, n = 5). (f) Immunohistochemical images of LOX-1 positive cardiomyocyte (Arrows indicates typical LOX-1-positive cells: brown staining.) in the hearts of mice treated with vehicle or DOX. Representative scaled photographic images are shown (scale bars: 20 μm).

As shown in the top panel of Fig 4A, the left ventricle cavity size in three groups of mice excluding WT mice treated with DOX was equivalent in horizontal sections of the hearts subjected to HE staining. The left ventricle cavity size of WT mice treated with DOX was larger than in the other three groups, which well correlated with the results of echocardiography. It indicated that DOX-induced LV dilatation was attenuated in LOX-1 KO mice compared with WT mice. As shown in the left panel of Fig 4B, we measured the heart to body weight ratio of all four groups at 14 days after DOX treatment. These ratios among the four groups were unaffected by DOX treatment.

Next, we performed quantitative assessments of cardiomyocyte diameter using a multipurpose image processor. Twenty cardiomyocytes/heart (four groups of eight hearts) were randomly selected and then the average transverse diameter of each cardiomyocyte was calculated as a quantitative analysis of cardiomyocyte size. Cardiomyocyte size decreased by about 10% in WT mice treated with DOX compared with that treated with vehicle (Fig 4B, center). There was no difference in cardiomyocyte size between LOX-1 KO mice treated with DOX and those treated with vehicle, as shown in Fig 4B. Interestingly, as shown in Fig 4A (middle), a microscopic image of WT mice treated with DOX indicated atrophic degeneration of cardiomyocytes.

Fig 4A shows representative microscopic images of Sirius Red staining, which indicates the degree of myocardial fibrosis. Hearts from four groups (eight hearts each group) were stained with Sirius Red, and 20 independent 400x magnified fields (/heart) were analyzed for quantitative analysis of % fibrosis. Cardiac tissue of WT mice treated with DOX exhibited more prominent collagen fiber content, which was dyed pink, in comparison with WT mice treated with vehicle (Fig 4B, right). On the other hand, Sirius Red staining of cardiac tissue of LOX-1 KO mice treated with DOX was significantly suppressed compared with that of DOX-treated WT mice (Fig 4A and 4B), which was at the same level as in LOX-1 KO mice treated with vehicle.

To evaluate which types of cells are important in DOX-induced cardiomyopathy, we examined the expression of LOX-1 in cardiomyocytes, endothelial cells, fibroblasts and inflammatory cells by immunofluorescence study, Western blotting and FACS. As shown in Fig 4C(a), microscopic images indicated strongly enhanced LOX-1 expression (Alexa Fluor 488; green) in cardiomyocytes treated with DOX compared with those treated with vehicle. Cardiomyocytes were identified with phalloidin (Alexa Fluor 555; red), which causes irreversible polymerization of actin into microfilaments. LOX-1 expression in endothelial cells and fibroblasts treated with DOX was not elevated compared with that treated with vehicle (Fig 4C(b) and 4C(c)). Endothelial cells were identified with anti-CD31 Ab (Alexa Fluor 555; red, Fig 4C(c)) and cardiac fibroblasts were identified with anti-Vimentin antibody (Alexa Fluor 555; red, Fig 4C(c)). We further performed Western blotting for the quantification of LOX-1 expression. As in the immunofluorescence study, LOX-1 expression was more abundant in cardiomyocytes treated with DOX than in those treated with vehicle and other cells treated with vehicle or DOX. On the other hand, LOX-1 expression was significantly lower in endothelial cells and fibroblasts treated with vehicle than cardiomyocytes treated with vehicle as a baseline (Fig 4C(d)). Moreover, endothelial cells and fibroblasts treated with DOX also expressed LOX-1 as much as those treated with vehicle (Fig 4C(d)). Interestingly, very small LOX-1 expression was observed in cardiac fibroblasts treated with either vehicle or DOX Fig 4C(d).

To examine whether or not inflammatory cells enhanced LOX-1 expression after DOX treatment, FACS analysis was performed. As shown in Fig 4C(e), DOX treatment did not increase LOX-1 expression in CD45 positive-inflammatory cells (neutrophils, monocyte and lymphocytes) in the peripheral blood of mice at all. This indicated that the expression of LOX-1 in CD45-positive inflammatory cells was smaller than that in cardiomyocytes in the heart treated with DOX, because the number of CD45-positive cells is not larger than that of cardiomyocytes as shown in Fig 3A.

Next, we evaluated LOX-1 expression in the hearts of mice treated with vehicle or DOX by immunohistochemistry. In Fig 4C(f), we confirmed that cardiomyocytes expressed LOX-1 in the hearts of mice treated with vehicle. Moreover, we found that administration of DOX strongly enhanced LOX-1 expression in cardiomyocytes of mouse hearts.

**Fig 5:**
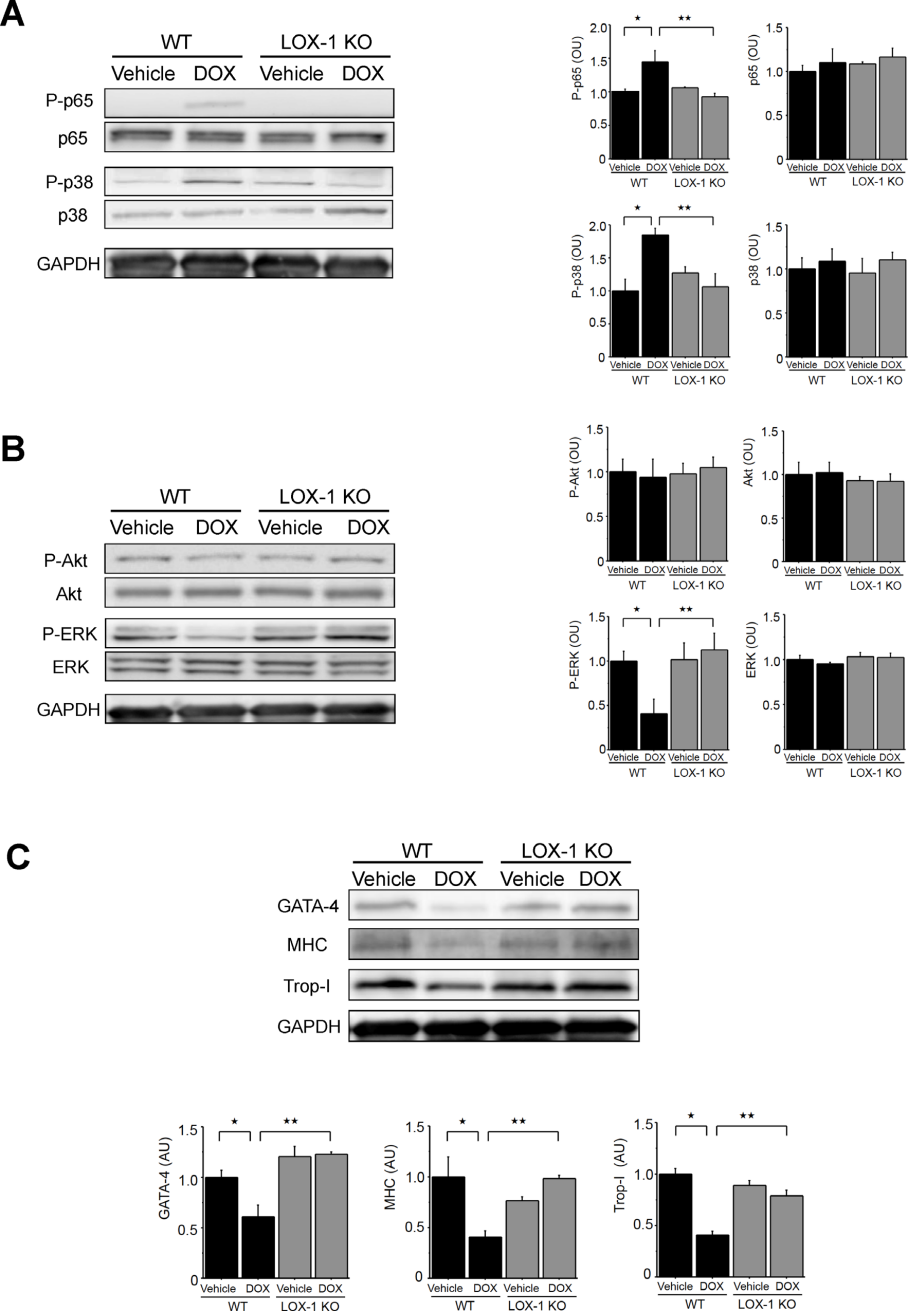
Signal transduction proteins of LOX-1 (phospho- and total NF-κB, MAPKs and Akt) and sarcomeric-related proteins (GATA-4, MHC and Trop-I). A: Representative Western blot and quantification of phospho- and total p65 (P-p65 and p65) and phospho- and total p38 (P-p38 and p38) in the four experimental groups. B: Representative Western blot and quantification of phospho- and total Akt (P-Akt and Akt) and phospho- and total ERK (P-ERK and ERK) in the four experimental groups. C: Representative Western blot and quantification of GATA-4, MHC and troponin I (Trop-I) in the four experimental groups. GAPDH is the internal control. Presented values (AU: arbitrary units) are mean±SEM. ^★^P<0.05, WT/Vehicle vs. WT/DOX, ^★★^P<0.05, WT/DOX vs. LOX-1 KO/DOX, n = 4; day14.

We performed Western blotting to evaluate cell signalings, including NF-κB, MAPKs and Akt, which are related to LOX-1 signaling. At first, we evaluated and quantified total and phosphorylated p65 subunit of NF-κB (P-p65) and p38 MAPK (Fig 5A). Although P-p65 was enhanced in the hearts of WT mice treated with DOX compared with that of WT mice treated with vehicle, P-p65 in the hearts of LOX-1 KO mice treated with DOX was significantly attenuated compared with that of WT mice treated with DOX. Total p38 MAPK remained constant and phosphorylated p38 MAPK (P-p38) was significantly enhanced in the hearts of WT mice treated with DOX compared with that of WT mice treated with vehicle. P-p38 in the hearts of LOX-1 KO mice treated with DOX was suppressed compared with that of WT mice treated with DOX. Phosphorylation of both Akt and ERK protein was analyzed, as shown in Fig 5B. There was no difference of total and phosphorylated Akt protein (P-Akt) between WT and LOX-1 KO mice that were treated with vehicle or DOX. Total ERK remained unchanged among the four experimental groups. Although phosphorylation of ERK (P-ERK) was significantly inhibited in the hearts of WT mice treated with DOX compared with that of WT mice treated with vehicle (Fig 5B), this inhibition of P-ERK was not observed in the hearts of LOX-1 KO mice treated with DOX. This phosphorylation level of ERK in the hearts of LOX-1 KO mice treated with DOX was similar to that of WT mice treated with vehicle, which is the basal level of phosphorylation.

Next, we quantified the expression of sarcomeric-related proteins as shown in Fig 5C. GATA-4 is a major transcription factor regulating sarcomeric genes such as cardiac MHC and Trop-I. GATA-4 expression was suppressed in the hearts of WT mice treated with DOX compared with that of WT mice treated with vehicle, but this suppression of GATA-4 was not observed in the hearts of LOX-1 KO mice treated with DOX. Cardiac sarcomeric proteins, MHC and Trop-I, were also subjected to quantitative comparisons among the four groups in this experiment. Western blot analyses showed that the expressions of MHC and Trop-I were downregulated in the hearts of WT mice treated with DOX compared with those of WT mice treated with vehicle. These two sarcomeric proteins were not suppressed in the hearts of LOX-1 KO mice treated with DOX in comparison with the levels in those of WT mice treated with vehicle as well as P-ERK and GATA-4 expression.

## Discussion

In the present study, we showed for the first time that LOX-1 plays an important role in the development of DOX-induced cardiomyopathy. The major evidence is that hearts of LOX-1 KO mice were protected against development of LV dysfunction, cardiomyocyte degeneration and interstitial fibrosis after DOX treatment. These actions of LOX-1 on DOX-induced cardiomyopathy were associated with adverse changes in ROS production, inflammatory infiltration, fibrosis and LOX-1-related signaling pathway in the hearts.

DOX, a typical anthracycline, is extremely effective against many tumors and widely used in the world but it is known to have adverse effects on various tissues via oxidative stress and it has been shown that the administration of DOX causes cardiomyopathy and fatal heart failure as described in the Introduction [24–26]. Unfortunately, no effective treatment for DOX-induced cardiomyopathy and heart failure is presently available. Therefore, we investigated whether or not LOX-1 deletion protects from the transition toward heart failure in response to DOX administration.

LOX-1 was first identified as an oxidized LDL receptor on endothelial cells, which induced endothelial dysfunction, atherosclerosis and angiogenesis, but the pathophysiological role of LOX-1 has not been fully understood in the cardiovascular disease yet [1–5, 7, 9, 10, 12, 40]. Although LOX-1 is also supposed to be an important player in heart diseases, physiological role of LOX-1 in heart failure by various mechanisms must be investigated aggressively [22].

At first, we generated DOX-induced cardiomyopathy, and the assessment of LV function by transthoracic echocardiography and Millar micro-tip catheter transducer showed that DOX administration resulted in a significant increase of LV cavity dilatation and LV dysfunction just within a week after DOX administration and this LV dysfunction continued to worsen progressively in the hearts of WT mice. Surprisingly, we found that LOX-1 KO mice did not present LV cavity dilatation and LV dysfunction like WT mice did after DOX treatment.

LOX-1 plays a critical role in ischemic heart failure. Mehta et al. revealed that LOX-1 deletion in mice improved cardiac function via cardiac remodeling, which started immediately following ischemic reperfusion [12, 36, 41]. It has been also reported that suppression of LOX-1 prevented cardiac remodeling in a rat model of myocardial ischemia–reperfusion and also reduced the size of myocardial infarct and improved left ventricular function by inhibiting apoptosis and lipid oxidation in cardiomyocytes [42]. Interestingly, serum levels of soluble LOX-1 were reported to be elevated significantly in patients with LV systolic dysfunction and hypertrophy, and were correlated with the decrease in EF in patients with ACS [17, 19, 23]. These also indicate that LOX-1 has a pathophysiological function in heart disease.

Here, our findings are the first suggesting that deletion of LOX-1 protects against DOX-induced cardiomyopathy. The mechanisms responsible for DOX-induced cardiomyopathy have also been studied here and highly suggestive data were obtained.

ROS sources include the mitochondrial electron transport chain, xanthine oxidase, dysfunctional NOS and NADPH oxidase [35]. Although DOX induced cardiomyopathy has been believed to occur primarily via generation of ROS in mitochondria [43, 44], Nox2 NADPH oxidase-derived ROS generation has been also reported to make a substantial contribution to DOX-induced cardiomyopathy [45]. LOX-1 activation also elevates ROS levels such as superoxide anions and hydrogen peroxide via activation of a membrane-bound NADPH oxidase [46–49]. In fact, we examined and found LOX-1 KO mice showed significant decrease in Nox2 expression and ROS production in ischemic limbs [20]. Nox2-derived ROS generation potentiates mitochondrial ROS generation in cardiomyocytes [50]. Although the mitochondria are thought to be the primary source of DOX-induced oxidants [24], Nox2-derived ROS generation is also supposed to be very important as well as mitochondrial ROS generation in DOX-induced cardiomyopathy. In this experiment, we found that DOX administration increases ROS in cardiac tissue of WT mice but not in that of LOX-1 KO mice.

On the other hand, ROS production caused by DOX administration is supposed to increase the production of peroxidation molecules such as oxidized LDL, which is the ligand of LOX-1. The production of oxLDL could result in LOX-1 activation, causing the further release of ROS in cardiac tissue consisting of various cardiac cells (cardiomyocytes, fibroblasts, endothelial cells and migrating leukocytes), which functionally communicate and cross-talk with each other [36, 47, 48, 51]. Interestingly, we found that LOX-1 expression in the heart tissues of WT mice were upregulated after DOX administration but not in the heart tissues of WT mice after vehicle administration. This suggests positive feedback between activation and upregulation of LOX-1. As LOX-1 also acts as a leukocyte adhesion molecule which is same as VCAM-1 [11, 14, 52], enhanced LOX-1 and VCAM-1 expressions in the DOX-treated hearts of WT mice are supposed to promote the infiltration of inflammatory cells into cardiac tissues collectively.

More ROS production derived from activation of LOX-1 results in phosphorylation of MAPKs and activation of NF-κB [47, 53]. In our experiment, ROS production was enhanced and p38 MAPK was phosphorylated in the hearts of WT mice treated with DOX. In contrast, both of them were suppressed in LOX-1 KO mice treated with DOX. NF-κB activation, which occurs sequentially after p38 MAPK phosphorylation [54], was observed in the hearts of WT mice treated with DOX, but not LOX-1 KO mice. This suggests that the upregulated expressions of NF-κB-inducible molecules, pro-inflammatory mediators such as TNF-α, IL-1ß, VCAM-1 and LOX-1 could result in more inflammation of cardiac tissue. Actually, TNF-α and IL-1ß were elevated in cardiac tissue of WT mice, but not in LOX-1 KO mice after DOX administration in our experiments. We suppose that the downstream effector of NF-κB: LOX-1, TNF receptor and IL-1 receptor cooperatively induce enhanced inflammation in the hearts after DOX administration, resulting in more leukocyte infiltration including CD45-positive cells.

ROS also plays important roles in the development of cardiac fibrosis under DOX toxicity [55]. Synergistic generation of ROS in the hearts treated with DOX is thought to upregulate expressions of TNF-α and IL-1ß, and induce accumulation of leukocytes and maybe also increase the TGF-β expression level, resulting in induction of cardiac fibrosis [12]. Therefore, we speculated that the deletion of LOX-1 under DOX treatment results in a significant reversal of DOX-induced cardiac fibrosis in our experiment, which could attenuate DOX-induced cardiomyopathy.

In DOX-induced cardiomyopathy, scattered vacuolated cardiomyocytes and patchy myocardial interstitial fibrosis are observed in optical microscope and electron microscope study also shows myofibrillar loss, vacuolization and extensive diffuse fibrosis [56–60]. These degenerations indicate DOX-induced cardiomyocyte atrophy and degeneration which are similar to those of dilated cardiomyopathy. It has been reported that GATA-4 (a key transcription factor related to regulation of cardiac protein expression) was downregulated in the hearts treated by DOX, which possibly cause cardiac atrophy [61]. In that report, they also speculated that activation of ERK by tadalafil attenuate DOX-induced cardiomyopathy. ERK directly phosphorylates Ser-105 in GATA-4 and dominant negative GATA-4 attenuated activated MEK1-induced myocyte growth, suggesting an important role of ERK in cardiac hypertrophy through GATA-4 [50]. Other investigators also reported that DOX inactivates ERK and its inactivation is associated with myofibrillar loss and reduced LV function [59, 62]. These findings strongly support our result that DOX caused a significant decrease of P-ERK1/2 and cardimyocyte atrophy in the hearts.

We found downregulation of GATA-4 transcription factor and regression of sarcomeric proteins such as MHC and Trop-I which are regulated by GATA-4 in the hearts of WT mice treated with DOX. DOX-induced cardiomyopathy was reversed by the addition of erythropoietin and this cardioprotective mechanism by erythropoietin is mediated by the phosphorylation of ERK [59]. Although our experiments also showed DOX treatment reduced the phosphorylation of ERK in WT mice after DOX treatment in a similar fashion [49], LOX-1 KO mice treated with DOX preserved the phosphorylation of ERK as well as WT mice treated with vehicle. Therefore, preserved ERK signaling prevents cardiomyocyte degeneration, which is not in coordination with Akt signaling. Because Akt activation and inactivation were not observed in our experiment, we have not yet elucidated the mechanisms by which deletion of LOX-1 restores ERK inactivation and cardiomyocyte degeneration. In this way, cardiomyocyte atrophy and degeneration by DOX treatment were attenuated trough ERK/GATA-4 pathway in LOX-1 KO mice.

Furthermore, we examined 1) the LOX-1 expression in the isolated cells (cardiomyocytes, fibroblasts and endothelial cells) treated with vehicle or DOX and inflammatory cells in the peripheral blood of vehicle-treated mice and DOX-treated mice and 2) the LOX-1 expression in the hearts of mice treated with vehicle or DOX. In these experimental results as described in the results section of this paper, LOX-1 was expressed mainly in cardiomyocytes and modestly in endothelial cells, fibroblasts and inflammatory cells in the heart treated with DOX. This result strongly indicates LOX-1 expressed in cardiomyocytes is the most important for DOX-induced heart failure, and that LOX-1 in endothelial cells, fibroblasts and inflammatory cells may also be involved in DOX-induced cardiomyopathy in some form because these cells also expressed LOX-1 modestly.

Our study provides a new insight into the pathophysiological role of LOX-1 in DOX-induced cardiomyopathy using LOX-1 KO mice. This study suggests that 1) the linkage of LOX-1 activation with subsequent p38 MAPK and NF-κB activation after DOX treatment causes cardiac inflammation and fibrosis, and ERK inactivation after DOX treatment leads to cardiomyocyte degeneration, including cardiomyocyte shrinkage and sarcomeric degeneration, 2) the deletion of LOX-1 rescued DOX-induced cardiomyopathy caused by cardiac inflammation, fibrosis and cardiomyocyte degeneration because LOX-1 signaling was previously described as being shut down and 3) LOX-1 is expressed mainly in cardiomyocytes and modestly in endothelial cells fibroblasts and inflammatory cells in the heart, indicating that LOX-1 expressed in cardiomyocytes plays the most important role in DOX-induced cardiomyopathy.

In our experiment, we could find the beneficial effect of LOX-1 deletion on lethal disease -DOX-induced cardiomyopathy- whose mortality is approximately 50% for the first time [60]. Our results indicate that the physiological suppression or deletion of LOX-1 is supposed to be a novel therapy against DOX-induced cardiomyopathy. This is the most important contribution and achievements of our experiments.

We need to investigate further the physiological role of LOX-1 in heart failure other than DOX-induced cardiomyopathy.

## Conclusions

We concluded that LOX-1 in cardiomyocytes plays the most important roles in the pathogenesis of DOX-induced cardiomyopathy because LOX-1 deletion results in the improvements in cardiac function, myocardial inflammation and fibrosis and degenerative changes of cardiomyocytes after DOX treatment via alteration of the LOX-1-related signaling pathway.
